# Health care ethics programs in U.S. Hospitals: results from a National Survey

**DOI:** 10.1186/s12910-021-00673-9

**Published:** 2021-07-29

**Authors:** Marion Danis, Ellen Fox, Anita Tarzian, Christopher C. Duke

**Affiliations:** 1 Department of Bioethics, National Institutes of Health, Building 10, Rm 1C118, Bethesda, MD 20892-1156 USA; 2 Fox Ethics Consulting, Arlington, VA 22213 USA; 3 National Center for Ethics in Health Care, Veterans Health Administration, 811 Vermont St. NW., Washington, DC 20571 USA; 4 Altarum Institute, 3520 Green Ct., Suite 300, Ann Arbor, MI 48105 USA

**Keywords:** Healthcare ethics, Ethics, institutional, Survey, Empirical research

## Abstract

**Background:**

As hospitals have grown more complex, the ethical concerns they confront have grown correspondingly complicated. Many hospitals have consequently developed health care ethics programs (HCEPs) that include far more than ethics consultation services alone. Yet systematic research on these programs is lacking.

**Methods:**

Based on a national, cross-sectional survey of a stratified sample of 600 US hospitals, we report on the prevalence, scope, activities, staffing, workload, financial compensation, and greatest challenges facing HCEPs.

**Results:**

Among 372 hospitals whose informants responded to an online survey, 97% of hospitals have HCEPs. Their scope includes clinical ethics functions in virtually all hospitals, but includes other functions in far fewer hospitals: ethical leadership (35.7%), regulatory compliance (29.0%), business ethics (26.2%), and research ethics (12.6%). HCEPs are responsible for providing ongoing ethics education to various target audiences including all staff (77.0%), nurses (59.9%), staff physicians (49.0%), hospital leadership (44.2%), medical residents (20.3%) and the community/general public (18.4%). HCEPs staff are most commonly involved in policy work through review of existing policies but are less often involved in development of new policies. HCEPs have an ethics representative in executive leadership in 80.5% of hospitals, have representation on other hospital committees in 40.7%, are actively engaged in community outreach in 22.6%, and lead large-scale ethics quality improvement initiatives in 17.7%. In general, major teaching hospitals and urban hospitals have the most highly integrated ethics programs with the broadest scope and greatest number of activities. Larger hospitals, academically affiliated hospitals, and urban hospitals have significantly more individuals performing HCEP work and significantly more individuals receiving financial compensation specifically for that work. Overall, the most common greatest challenge facing HCEPs is resource shortages, whereas underutilization is the most common greatest challenge for hospitals with fewer than 100 beds. Respondents’ strategies for managing challenges include staff training and additional funds.

**Conclusions:**

While this study must be cautiously interpreted due to its limitations, the findings may be useful for understanding the characteristics of HCEPs in US hospitals and the factors associated with these characteristics. This information may contribute to exploring ways to strengthen HCEPs.

## Background

Hospitals are complex organizations that grapple with numerous ethical issues related to patient care, population health, professional practice, employee relations, business relations, and organizational ethics. Depending on their ownership, mission, and affiliations, some hospitals also need to be concerned with government ethics, public administration ethics, faith-based ethics, research ethics, and the ethics of educating trainees.

Over the past 40 years, ethics scholars, professional organizations, and oversight organizations have recognized that the breadth of ethical issues that health care organizations face are not fully encompassed by exclusive attention to the clinician-patient relationship; they have called for more attention to the range of ethical issues that are faced by health care organizations [1–4].

U.S. hospitals have tended to address this wide range of ethical issues through an expanding patchwork of committees and offices. For example, within a given hospital, clinical ethics issues are often handled by an ethics consultation service or ethics committee while business and management issues are handled by compliance officers and human resources staff and research ethics issues are handled by an institutional review board (IRB). These entities tend to operate in relative isolation from each other, instead of working together to identify and address areas of overlap and gaps [5].

Yet in more recent years, there has been a call for U.S. health care institutions to move beyond the traditional patchwork model to adopt a unified programmatic approach that integrates the various “subspecialties” of health care ethics such as clinical ethics, organizational ethics, and research ethics [6]. The American Society for Bioethics and Humanities (ASBH) has explicitly endorsed “the trend toward integrating ethics across all subspecialties in an organization” [6]. One prime example of such an integrative approach to ethics is the IntegratedEthics™ model from the U.S. Department of Veterans Affairs National Center for Ethics in Health Care [5], which replaces the traditional, siloed, ethics committee approach with a single, overarching ethics program that coordinates and manages the organization’s provision of ethics services across the full range of content domains. Other ethics models that address a wide range of ethical issues that health care organizations encounter include the Southern California region of Kaiser Permanente, whose bioethics program integrates with many programs including quality management and compliance [7], and the Catholic Health Association (CHA)/Ascension model [8], which integrates with leadership and numerous institutional committees such as human resources (IRB), and patient relations. The cross-cutting and comprehensive nature of these programs is reflected in their mission statements. For example, the aim of the CHA/Ascension model is “to promote and support the identity and integrity of an organization and those within it; and the aim of the VA’s IntegratedEthics model is “to support, maintain, and improve ethics quality in health care” at three levels. Notably, none of these integrated ethics programs focuses exclusively on clinical ethics (i.e., the identification, analysis, and resolution of values conflicts or uncertainties that arise in the provision of health care in clinical settings) [9, 10]. Rather, they all address other ethics issues, such as those relating to resource allocation, advertising, relationships with employees, and human subjects research. Nor do these programs focus on individual decisions and actions alone; they are also concerned with the organizational systems, processes, environment, and culture [5].

Integrated ethics programs are “integrated” in three ways. First, whereas in many hospitals, ethics-related activities are carried out by various individuals and programs (such as ethics committees, leadership, and IRBs) in relative isolation from each other, integrated ethics programs have an integrative “umbrella” structure—i.e., a unified, coordinated programmatic approach. Second, whereas traditional ethics support services tend to focus on specific content areas, such as clinical ethics or research ethics, integrated ethics programs are comprehensive or at least very broad in scope, including many if not all types of ethical issues facing the organization. Third, whereas traditional ethics support services tend to focus on a narrow range of activities (e.g., ethics consultation, staff education, and policy review), integrated ethics programs employ many different strategies to accomplish their goals and integrate with other programs and individuals throughout the organization.

Despite the recent trend toward integrated programmatic approaches to ethics in health care organizations, prior empirical studies of health care ethics in U.S. hospitals have focused on a single programmatic structure, activity, or model. For example, there have been studies of clinical ethics consultation services [11–14], hospital ethics committees [15–18], clinical ethics policy [19], ethics and compliance programs [20], research ethics committees [21], and research ethics consultation [22]. But to our knowledge, no prior study has examined the full range of officially sanctioned entities within a hospital that support health care ethics—in other words, health care ethics programs (HCEPs).

To address this gap in knowledge regarding hospital-based HCEPs in the US, we included questions regarding ethics programs in a national survey of ethics consultation in US hospitals [23]. Because ethics consultation services are often part of HCEPs, certain characteristics of ethics consultation services cannot be meaningfully studied without studying the broader ethics program. For example, budgets and staffing are often allocated to ethics programs, to cover various ethics-related activities, and not to ethics consultation services separately. While the survey was largely focused on ethics consultation, this report examines the broader context of HCEPs beyond ethics consultation.

## Methods

The broader study of which this analysis is a part [23] replicates many of the methods from a prior national study of health care ethics consultation in U.S. hospitals [11]. A full description of the methods is provided in the publication of the broader study and is accompanied by the survey instrument [23].

### Survey instrument

A HCEP was defined in the survey questionnaire as follows:

For this survey, health care ethics program is defined as an officially sanctioned entity within a hospital that supports health care ethics by providing ethics-related services such as ethics policy development or ethics education. Services may be performed by one or more designated individual(s), committee(s), office(s), or other organizational structure(s). A health care ethics program may or may not provide ethics consultation.

This definition was intended to include all organized programmatic health care ethics activities regardless of how they were labeled, performed, or structured. To reinforce the idea that a HCEP is not necessarily limited to clinical ethics but can encompass a variety of other content areas, we began the survey with a question about scope that immediately followed the definition of a HCEP. Specifically, we gave a list of ethics-related functions (clinical ethics, business ethics, research ethics, regulatory compliance, ethical leadership, other) and asked respondents to select which functions their HCEP included.

Next respondents were asked a series of multiple-choice questions about the education activities, policy activities, and other activities of their HCEP. To examine their educational role, they were asked to select the target audiences to which their HCEP was responsible for providing ongoing ethics education. To examine their policy work, they were asked whether their HCEP participates in leading and/or assisting others in the development of new policies and/or the periodic review of existing policies. Regarding other activities, they asked whether their HCEP includes an ethics representative positioned at the executive leadership level in the organization, provides ethics representation to other hospital committees, leads large-scale quality improvement initiatives related to ethics, and is actively engaged in community outreach.

Respondents were also asked about their HCEP’s staffing, work hours, financial compensation of staff, source of funding, and reporting relationship through numeric response, numeric range, and multiple-choice questions. To characterize the staffing of HCEPs, respondents were asked how many individuals in total performed either paid or unpaid work for the hospital’s HCEP in the last year (including ethics consultation and other ethics activities). Respondents were then asked to estimate the average number of hours per week that each of these individuals devoted to HCEP work in the last year (< 1, 1–4, 5–9, 10–19, 20–29, 30–39, or ≥ 40 h/week). They were also asked how many of these individuals who worked for the HCEP received salary support (or equivalent financial compensation such as a consulting fee or a dedicated percentage of their salary) specifically for HCEP work. Respondents were then asked to estimate the total number of FTEs in salary support (or equivalent financial compensation) provided for HCEP work at their hospital. FTE was defined as follows: “FTE stands for Full-Time Equivalent. 1.0 FTE means the equivalent of one full-time salary; 0.1 FTE means the equivalent of 4 h per week.” Respondents were asked to provide an estimate even if they did not know the exact number. To determine the source of funding, respondents were asked to indicate how this salary support (or other financial compensation) for HCEP work was funded by estimating the percentage for each of the following funding categories: hospital, multi-hospital health care system that includes the hospital, university or school, patient billing, or other.

To determine the HCEP’s reporting relationship, respondents were asked which hospital administrator or senior leader had oversight responsibility for health care ethics. To clarify the meaning of oversight responsibility the following explanation was provided: “This person is not the person who leads or manages the ethics program, but rather a person at a higher leadership level in the hospital. This person would typically receive periodic reports about the ethics program and would potentially intervene if there were big problems with the program.” To further characterize the program’s relationship with this hospital administrator, respondents were asked to rate on a scale of 0 to 10 the administrator’s level of awareness about the hospital’s health care ethics activities.

Finally, to explore the challenges faced by HCEPs, respondents were asked to give detailed and specific answers to the following open-ended questions: What do you think is the #1 greatest challenge relating to the hospital’s health care ethics program right now? What do you think would help the hospital to overcome or manage that challenge? The full survey is published elsewhere [23].

Respondents in hospitals that reported not having an ethics consultation service were asked, “Is there some other individual, committee, office or other structure within your hospital that supports health care ethics by providing ethics-related services such as ethics policy development or ethics education?” These hospitals, which were considered to have a HCEP, were invited to take a different version of the survey that included 23 of the primary questions from the full survey^1^ and seven contingency questions from that survey.^2^

### Survey sample

The survey sample was drawn from the pool of all general hospitals that participated in the 2016 American Hospital Association Annual Survey of Hospitals [24]. The random sample, stratified based on bed size category, included 600 hospitals. Survey respondents at each hospital were identified and contacted using a standardized protocol and script as described elsewhere [23].

### Analysis

Data were analyzed using SAS, version 9.3*.* Data were weighted by bed size category to adjust for the stratified sample using the degrees-of-freedom method to make inferences about the entire population of U.S. general hospitals [25]; Descriptive statistics were used to describe survey measures. We used a series of one-way ANOVAs with contrasts and chi-squares to evaluate the associations between hospital characteristics and specific survey measures. All contrasts used the Scheffé method of adjustment for multiple comparisons [26]. We used a two-sided probability of 0.05 as the criterion for statistical significance. Results are presented as weighted percentages extrapolated from completed responses to the survey.

In addition to analyzing the survey responses, we calculated the number of content areas that were included within the scope of the hospital’s HCEP (possible score range 0–5), the number of educational target audiences to which the HCEP was responsible for providing ongoing ethics education (possible score range 0–7), the number of types of policy work in which the HCEP participated (possible range 0–4), and the number of other activities performed by the HCEP (possible range 0–4). We also constructed a variable for “total workload,” which was estimated from the aggregated number of person-hours per week that were devoted to (paid or unpaid) HCEP work in the last year by all of the individuals who performed work for the hospital’s HCEP combined. For each of the numeric ranges specified for the average number of hours per week devoted to HCEP work by individuals, we multiplied the number of individuals whose hours per week fell in that range by the midpoint in that range, then summed the results across all numeric ranges. For example, if a hospital indicated that 1 person devoted between 5 and 9 h per week and a second person devoted ≥ 40 h per week, the total workload would be calculated as follows: (1 × 7) + (1 × 40) = 47 h per week.

Some responses to questions regarding average hours per week, number of individuals who received financial compensation, and FTEs did not seem plausible as explained below. For these questions, one author reviewed results to identify potentially implausible responses; these were eliminated if two other authors agreed with doing so. We report results both with and without these implausible responses.

#### Content analysis

Responses to open-ended questions regarding the HCEP’s greatest challenge and proposed strategies to overcome this challenge were coded using content analysis as follows. Following a reading of responses, a coding scheme was developed by a research assistant. It was then reviewed and revised by one of the authors. Codes were then assigned to responses by these two individuals and reviewed by a second author. Any disagreement regarding assigned codes was reconciled by deliberation. The weighted percentage of respondents in each hospital category who provided open-ended responses matching the assigned codes was calculated. Examples of open-ended responses were selected for inclusion in this manuscript as illustrative of these codes.

## Results

### Study hospitals

Among the 600 sampled hospitals, one closed before data collection; 462 participated and completed all or part of the study for a response rate of 77.1%. Among these participating hospitals, 438 had an ethics consultation service and were eligible for the version of the online survey designed for hospitals with ethics consultation services; 365 completed some or all of this online survey. An additional 16 of the 462 participating hospitals had a HCEP but no ethics consultation service so were eligible for the modified version of the online survey; of these 16, seven completed at least one question on that survey, but only one hospital answered any of the questions reported in this paper beyond reporting that they had a HCEP. The remaining eight of the 462 participating hospitals had no ethics consultation service and no HCEP so were not eligible for either online survey. Here we report data regarding the prevalence of HCEPS based on the 462 participating hospitals, and data about the characteristics of HCEPs based on the 372 hospitals that completed all or part of either online survey. There were no significant differences between sampled hospitals and participating hospitals, or between hospitals that took the online survey and all participating hospitals, for any of the hospital characteristics, as reported elsewhere [23].

### Prevalence of HCEPs

Based on participants’ responses, an estimated 97.1% of hospitals had a HCEP. The prevalence of HCEPs did not vary significantly by hospital bed size, ownership category, academic affiliation, or urban/rural location (see Table 1).

**Table 1:** Prevalence and Scope of Health Care Ethics Programs (HCEPs) in US General Hospitals

Hospital category	Population estimate (% of hospitals)					
	Hospital has a HCEP (N = 462)	Content areas included in the scope of HCEPs (N = 291)				
		Clinical ethics	Business ethics	Research ethics	Regulatory compliance	Ethical leadership
*Bed size*						
1–99 *(reference category)*	96.0	100	16.7	0	27.8	27.8
100–199	97.4	90.9	36.4	13.6	27.3	34.1
200–299	98.8	96.2	36.5	23.1	38.5	50.0
300–399	98.5	100	31.1	26.7**	33.3	46.7
400–499	97.7	97.0	30.3	36.4**	18.2	60.6
500 +	99.3	97.0	25.3	35.4**	28.3	36.4
*Ownership*						
Govt. (Federal)	100.0	97.0	66.2	19.8	29.8	93.9
Govt. (non-Federal)	98.3	96.6	37.7	3.9	16.0	43.4
Investor-owned; for-profit	97.5	96.8	15.0	3.8	26.8	27.6
Nongovt. (not-for-profit)—church operated	99.7	97.7	26.6	15.8	31.1	40.6
Nongovt. (not-for-profit)—other	95.4	96.9	22.0	16.0	33.5	29.2
*Academic affiliation*						
Major teaching	100.0	98.9	33.1	44.8**	33.8	52.2
Minor teaching	99.5	96.6	31.6	17.8**	32.2	50.2
Non-teaching *(reference category)*	95.6	97.1	21.8	5.2	26.4	24.2
*Location*						
Urban	98.8	96.6	30.5	27.4*	29.7	44.7
Rural *(reference category)*	95.0	96.0	24.0	16.0	28.0	16.0
Total	97.1	97.0	26.2	12.6	29.0	35.7

*p < .01

**p < .0001

### Scope of HCEPs

In almost all hospitals (97.0%), the scope of the hospital’s HCEP included the clinical ethics functions of the hospital. In a minority of hospitals, the HCEP scope included the hospital’s ethical leadership functions (35.7%), regulatory compliance functions (29.0%), business ethics functions (26.2%), and research ethics functions (12.6%). The only significant difference across hospital categories with regard to these functions was the percentage of hospitals whose HCEPs included research ethics, as shown in Table 1.

The mean number of content areas included in the scope of hospitals’ HCEPs was 2.3 (median 2, range 0 to 5). The HCEP included one content area in 43.6% of hospitals, two in 25.1%, three in 18.2%, four in 6.7%, and all five in 5.1%. The mean number of content areas varied significantly based on level of academic affiliation and hospital location (see Table 2).

**Table 2:** Scope and activities of HCEPs

Hospital category	Breadth of scope	Breadth of activities		
	Ethics content areas	Target educational audiences	Policy work	Other activities
*Bed size*				
1–99 *(reference category)*	1.7	2.8	2.3	1.4
100–199	2.0	3.3	2.3	1.7
200–299	2.4**	2.7	2.4	1.8
300–399	2.4	3.3	2.6	1.8
400–499	2.4	4.2	3.2	2.2
500 +	2.2	3.7	2.8	1.8
*Ownership*				
Govt. (federal) *(reference category)*	3.1	2.0	2.9	2.0
Govt. (non-Federal)	2.0	3.1	2.2	1.3
Investor-owned; for-profit	1.7	2.4	1.7	1.5
Nongovt. (not-for-profit)—church operated	2.1	3.5	2.6	1.8
Nongovt. (not-for-profit)—other	2.0	3.1	2.5	1.7
*Academic affiliation*				
Major teaching	2.5****	4.0*	3.1****	2.0***
Minor teaching	2.3****	3.5*	2.8****	1.8***
Non-teaching *(reference category)*	1.7	2.7	2.1	1.4
*Location*				
Urban	1.8**	2.7*	2.7****	1.9****
Rural *(reference category)*	2.1	3.3	1.8	1.1
Total	2.3	3.1	2.4	1.6

*p < .05

**p < .01

***p < .001

****p < .0001

### Activities of HCEPs

HCEPs were responsible for providing ongoing ethics education to the following target audiences: all staff in 77.0% of hospitals, nurses in 59.9% of hospitals, staff physicians in 49.0% of hospitals, leadership/management in 44.2% of hospitals, other non-clinical staff in 40.2% of hospitals, medical residents in 20.3% of hospitals, and the community/general public in 18.4% of hospitals. These percentages did not vary significantly across hospital categories except for medical residents and staff physicians as shown in Table 3. The HCEP was responsible for one target audience in 36.8% of hospitals, two or three in 18.3%, four or five in 29.9%, and six or seven in 14.0%. The mean number of target audiences was 3.1 (median 3, range 1–7). The number of target audiences varied significantly based on level of academic affiliation and hospital location (see Table 2).

**Table 3:** Target audiences to which health care ethics programs have responsibility for providing ongoing ethics education (N = 278)

Hospital category	Population estimate (% of hospitals)						
	All staff	Leadership/ manage-ment	Staff physicians	Medical residents	Nurses	Non-clinical staff	Community/general public
*Bed size*							
1–99 *(ref. category)*	88.2	47 .1	41.2	.00	58.8	35.3	11.8
100–199	61.5	46.2	59.0	28.2**	61.5	51.3	25.6
200–299	74.5	33.3	39.2	25.5**	54.9	29.4	11.8
300–399	66.7	40.0	53.3	40.0**	55.6	42.2	28.9
400–499	83.9	61.3	71.0	54.8**	77.4	48.4	25.8
500 +	67.4	35.8	65.3	62.1**	64.2	48.4	31.6
*Academic affiliation*							
Major teaching	67.2	35.1	67.7**	70.9**	73.3	53.7	28.2
Minor teaching	71.2	52.3	64.9**	31.7**	70.8	45.9	16.5
Non-teaching *(ref. category)*	82.3	39.9	35.5	5.3	50.5	34.3	18.3
*Location*							
Urban	70.1	40.9	58.3*	45.3**	62.6	43.7	24.4
Rural *(ref. category)*	79.2	41.7	41.7	12.5	54.2	41.7	29.2
Total	77.0	44.2	49.0	20.3	59.9	40.2	18.4

*p < .01

**p < .0001

HCEPs participated in the following types of policy work: leading the development of new policies (52.2%), assisting others who were leading the development of new policies (57.4%), leading periodic review of existing policies (75.5%), and assisting others who were leading the periodic review of existing policies (55.1%). These percentages varied based on hospitals characteristics as shown in Table 4. The mean number of types of policy work was 2.4 (median 2, range 1–4). The HCEP participated in one type of policy work in 26.2% of hospitals, two types in 35.2%, three types in 10.9% and all four types in 27.7%. This number of types of policy work varied significantly based on level of academic affiliation and hospital location (see Table 2).

**Table 4:** Policy activities and other activities of health care ethics programs

Hospital category	Population estimate (% of hospitals)							
	Policy activities (N = 276)				Other activities (N = 244)			
	Lead policy develop-ment	Assist policy develop-ment	Lead policy review	Assist policy review	Represen-tative in executive leadership	Represen-tative on other committees	Lead large-scale ethics QI initiatives	Actively engaged in community outreach
*Bed size*								
1–99 (ref. category)	50.0	43.8	81.3	50.0	92.9	21.4	7.1	14.3
100–199	41.0	66.7	71.8	51.3	65.7***	54.3***	22.9	25.7
200–299	56.3	54.2	68.8	56.3	88.9	40.0	26.7	26.7
300–399	64.4	68.9	71.1	60.0	63.2	63.2***	26.3	28.9
400–499	77.4	87.1	80.6	71.0	75.0	75.0***	32.1	39.3
500+	59.8	79.4	68.0	76.3	63.1	60.7***	25.0	33.3
*Academic affiliation*								
Major teaching	69.1	90.2***	74.3	71.6	56.7*	84.0***	32.4***	29.8
Minor teaching	57.7	74.9***	79.1	64.9	79.9	43.0	28.5***	29.2
Non-teachingref. category)	45.8	40.3	73.2	45.8	84.6	32.2	6.9	16.2
*Location*								
Urban	59.7	72.3***	71.9***	66.4***	70.3	58.6**	27.0***	31.1
Rural (ref. category)	47.8	47.8	65.2	43.5	81.8	27.3	4.5	18.2
Total	52.2	57.4	75.5	55.1	80..5	40.7	17.7	22.6

*p < .05

**p < .01

***p < .0001

HCEPs engaged in a variety of other activities besides education and policy work. The HCEP included an ethics representative positioned at the executive leadership level in the organization in 80.5% of hospitals, provided ethics representation to other hospital committees in 40.7% of hospitals, was actively engaged in community outreach in 22.6% of hospitals, and led large-scale quality improvement initiatives relating to ethics in 17.7% of hospitals. These activities varied based on hospital characteristics and shown in Table 4. The mean number of other activities was 1.6 (median 1, range 1–4). The HCEP performed one other activity in 61.5%, two other activities in 19.7% of hospitals, three other activities in 14.7%, and four other activities in 4.1%. The number of other activities varied significantly based on level of academic affiliation and hospital location (See Table 2).

### Staffing, workload, and compensation

The mean number of individuals who performed work (paid or unpaid) for HCEPs in the prior year was 11.0 (range 0–110, median = 8). The mean number of individuals who spent, on average, less than 1 h per week was 8.0; the number who spent 1–4 h per week was 3.0, the number who spent 5–9 h per week was 0.4, the number who spent 10–19 h per week was 0.3, the number who spent 20–29 h per week was 0.2, the number who spent 30–39 h per week was 0.05, and the number who spent 40 or more hours per week was 0.1. Only 5.9% of hospitals had one or more individuals who worked full-time for the HCEP; 4.9% had one, 0.3% had two, 0.2% had three, 0.4% had 4, and 0.3% had 5. The total number of individuals who performed work for HCEPs varied based on hospital characteristics as shown in Table 5.

**Table 5:** Staffing, workload, and financial compensation for health care ethics programs (HCEPs)

Hospital category	Mean number			
	Individuals who performed HCEP work in the last year (paid and unpaid) N = 269	Workload (Person-hours/week) of individuals who performed HCEP work N = 251	Individuals who received financial compensation specifically for HCEP work N = 228§	Estimated FTEs (full-time equivalents) provided for HCEP work N = 188§
*Bed size*				
1–99 *(ref. category)*	6.6	5.6	0.1	0
100–199	10.0	19.8	0.7	0.1
200–299	15.2****	50.8	1.1	0.1
300–399	18.9****	39.2	0.7	0.1
400–499	15.5	76.3	1.3	0.7
500 +	20.8****	104.0****	2.1***	1.0****
*Academic affiliation*				
Major teaching	19.3****	109.6****	2.1**	1.0****
Minor teaching	13.9****	29.6	0.6	0.2
Non-teaching *(ref. category)*	7.9	17.3	0.5	0
*Location*				
Urban	17.1****	41.8**	0.9**	0.2*
Rural *(ref. category)*	9.4	6.6	0.1	0
Total	11.0	29.1	0.6	0.2

*p < .05

**p < .01

***p < .001

****p < .0001

^§^Analysis performed after implausible answers were removed

The mean calculated total workload—i.e., the total number of person-hours per week devoted to paid or unpaid HCEP work in the last year by all the individuals who performed work for the HCEP combined—was 29.1 person-hours (range 0.5–1595.0, median = 9). Differences across hospital categories are shown in Table 5.

The mean number of individuals who received salary support or equivalent financial compensation such as a consulting fee or a dedicated percentage of their salary specifically for HCEP work was 1.6 (range 0–21, median = 0). In 61.6% of hospitals, the respondent indicated that no individuals received financial compensation specifically for HCEP work; in 16.7% of hospitals, one individual received compensation specifically for ethics; in 6.5% of hospitals two individuals received compensation specifically for ethics; in 5.0% of hospitals, three received compensation specifically for ethics; and in 10.2% of hospitals, four or more individuals received compensation specifically for ethics.

The mean estimate for the total number of FTEs in salary support or equivalent financial compensation provided to these individuals for HCEP work was 0.3 (range 0–15, median = 0). The estimated total FTE was 0 in 76.3% of hospitals, between 0 and 1.0 in 17.1% of hospitals, between 1.1 and 2.0 in 4.5% of hospitals, and 3 or more in 2.1% of hospitals.

In some cases, responses to the questions pertaining to these last three variables—workload, the number of individuals receiving financial compensation for HCEP work, and the total number of FTE—were deemed implausible, either because the responses were extreme outliers, or because the responses to these questions were inconsistent with each other. An example of an outlier response is one hospital where 42 individuals were reported to each spend between 20 and 29 h per week on HCEP work, while no individuals spent fewer than 20 h or more than 29 h per week. An example of an inconsistent response is one hospital where seven people were reported to each spend less than one hour per week on HCEP work and no one spent more than one hour per week, yet one person received salary support specifically for HCEP work and the total FTE for HCEP work was estimated to be two.

Removing such implausible responses had no effect on the results for workload. However, for both the number of individuals receiving financial compensation and for total estimated FTE, the results changed somewhat once implausible responses were removed. Specifically, for the number of individuals receiving financial compensation, for 23 of 251 respondents, responses were deemed implausible. Once these implausible responses were removed (N = 228), the results were as follows. The mean number of individuals who received salary support or equivalent financial compensation such as a consulting fee or a dedicated percentage of their salary specifically for HCEP work was 0.6 (range 0–21, median = 0). In 71.1% of hospitals, no individuals received financial compensation specifically for HCEP work; in 16.0% of hospitals, one individual received financial compensation; in 7.2% of hospitals two received compensation; in 2.4% of hospitals, three received compensation; and in 3.3% of hospitals, four or more individuals received compensation. The number of individuals receiving financial compensation varied based on bed size, academic affiliation, and urban/rural location as shown in Table 4, which summarizes the data after implausible answers were removed.

For estimated FTEs, 17 of 204 responses were deemed implausible. Based on the plausible responses only (N = 188), the mean estimated FTE was 0.2 (range 0–7, median = 0). Estimated FTE was 0 in 83.0% of hospitals, between 0.001 and 1.0 in 11.4% of hospitals, between 1.1 and 2.0 in 3.8% of hospitals, and 3 or more in 1.4% of hospitals. Estimated FTE varied based on bed size, academic affiliation, and location as shown in Table 5.

When we analyze the staffing and workload of HCEPs and extrapolate from responses regarding the total number of individuals who performed paid or unpaid work for hospitals, we estimate that, in aggregate, approximately 32,000 individuals performed work for US hospital HCEPs in the year prior to the survey.

In hospitals where salary support or other financial compensation was provided for HCEP work, it was funded by the hospital in 72.9% of hospitals, by a multi-hospital health care system that includes the hospital in 23.3% of hospitals, by a university or school in 0.9% of hospitals, through patient billing in 0.4% of hospitals, and by some other source for 2.5% of hospitals.

### Reporting relationship

The hospital administrator or senior leader who had oversight responsibility for health care ethics was the Chief Executive Office in 28.4% of hospitals, the Chief Medical Officer in 23.8% of hospitals, the Chief Nursing Officer in 18.7% of hospitals, the Chief Operating Officer in 4.7% of hospitals, and some other individual in 24.4% of hospitals. The mean rating for the hospital administrator’s level of awareness about the hospital’s health care ethics activities was 8.1 on a scale from 0 to 10 (where 0 = not at all aware and 10 = extremely aware).

### Greatest challenges facing ethics programs

Of the 372 respondents who completed some part of the online survey, 232 wrote in responses describing their HCEP’s #1 greatest challenge. The greatest challenges fell into 5 categories: resource shortages (including time, money, staff, recruitment, and training); underutilization of the ethics support services (i.e., staff were unaware of the service, did not understand the role of the service, did not appreciate its possible benefits, or did not identify a need for the service); lack of clarity about the ethics program’s scope, goals or purpose; lack of support from organizational leaders; and other challenges. See Table 6 for a summary of the percentages of hospitals reporting these challenges and sample quotes for each category. Hospitals with 1–99 beds were much more likely to report underutilization (50%) as their greatest challenge than hospitals with 500 + beds (16.5%) (*p* < 0.01). Similarly, non-teaching hospitals (44.3%) and rural hospitals (38.9%) were much more likely to report underutilization as their greatest challenge than major teaching hospitals (9.0%), and urban hospitals (19.6%) (*p* < 0.0001). Meanwhile, large hospitals, major teaching hospitals, and urban hospitals were more likely to list resource shortages as their greatest challenge (63.3% of hospitals with 500 + beds vs. 42.9% of hospitals with 1–99 beds; 69.5% of major teaching vs. 47.9% of non-teaching hospitals; 55.6% urban vs. 44.4% rural hospitals), although the differences for resource shortages were not statistically significant. While resource shortages was the most commonly reported greatest challenge overall, underutilization was the most commonly reported greatest challenge in hospitals with fewer than 100 beds.

**Table 6:** The #1 greatest challenges faced by health care ethics programs (HCEPs) (N = 232)

Type of challenge	% of hospitals^a^	Illustrative quotes
Resource shortages (time, money, staff, recruitment, and training)	48.5	Lack of time and training of some committee members, especially the physician members As awareness about our services grows the demand grows too, but without additional financial support we are unable to meet the demand. Additionally, while we have targeted known high-need areas, without further financial support we are unable to educate the institution more broadly or assess needs and develop initiatives to target other areas Not enough hours in the day/personnel to be present across the house
Underutilization of HCEP services	34.0	Lack of interest in ethics unless it is to back up a doctor who is making a difficult decision. There is a sense that attendings don't know why an ethics consultant would ever question an attending's decision, even though attendings don't always see how ethically complicated a decision might be
Other	22.1	Relationship building Making ethics a priority alongside issues of improving quality metrics
Lack of clarity about the HCEP’s goals or purpose	4.6	Defining/delineating scope with overlapping services (e.g., social work, palliative care) Clarification between clinical bioethics and “ethics and compliance (ECO)" related issues
Lack of support for the HCEP from organizational leadership	3.8	For me, it is the notion of buy in, whether financially, personnel resourcing, commitment from Senior Leadership. Currently, Healthcare Ethics at our Institution seems to be viewed as “oh it's nice to have, but we're not going to commit the resources to really developing and strengthening it.”

^a^Percentages are population estimates determined by weighting the sampling adjustments. Because responses were given multiple codes when they illustrated more than one type of challenge, percentages exceed 100%

### Strategies for overcoming challenges

Responses regarding what the respondent thought would help their hospital overcome or manage their greatest challenge fell into 8 categories: additional resources (for more time, staff, or other needs); training for staff; increased leadership buy-in; publicity or marketing; quality improvement efforts; data to demonstrate the value of the program; regional or national support or mandate; or some other solution that fell outside of these categories. See Table 7 for percentages of hospitals reporting each of these categories and sample quotes. Government hospitals were more likely to mention training as a solution than were for-profit hospital (Federal government (0.45), non-federal government (0.62), for-profit (0.18), *p* < 0.01).

**Table 7:** Proposed strategies for overcoming challenges of health care ethics programs (HCEPs) (N = 232)

Type of Strategy	% of hospitals^a^	Illustrative quotes
Training for existing staff	37.2	Webinars on types of ethical issues in rural facilities Online training Internal training or hire a lead clinical ethicist Perhaps education of medical staff on the real components of an ethics consult and the procedures in place that govern our work
Funds for additional time, staff, or other needs	30.3	More budgetary support for clinical ethics Our efforts to make a case for increased funding include … ongoing conversations with administration; most recently exploring philanthropic means of funding. It really comes down to funding
Other	29.5	Stabilizing a team will require leadership that is willing to make changes that unify the group to a shared sense of purpose Set up triggers for ethics consults It would help if physicians involved in ethics cases took ethics committee recommendations seriously, when the recommendations conflicted with their own inclinations
Increased leadership buy-in	9.4	A strategic plan for Bioethics at (Hospital) presented to the Hospital Board. We should be responsible for an annual report to the board as well
Data demonstrating value of HCEPs	8.0	Refrain from evaluating the quality of ethics services according to patient satisfaction, length of stay, or other ‘traditional’ quality metrics The ability to make a sound business case to get the ethicist position approved
Publicity/marketing	6.0	Having or developing a Marketing tool promotion of awareness of the ethics committee functions
Regional/national support or mandate for HCEPs	5.5	There is a (state) network that we are aware of and somewhat connected to, but we are not “members” of per se Mandates by regulatory and accreditation agencies National norms for adequate support
Quality assurance/quality improvement	5.2	development of quality measures to assess the benefit of clinical ethics consultation quality review of current consult and a formalized process for curbside consults Making demonstration of ethics knowledge a performance standard

^a^Percentages are population estimates determined by weighting the sampling adjustments. Because responses were given multiple codes when they illustrated more than one type of challenge, percentages exceed 100%

## Discussion

This nationwide survey of a random sample of US hospitals serves to describe the characteristics of HCEPs in US hospitals beyond ethics consultation. The characteristics we studied were: prevalence, scope, activities (education, policy, other activities), staffing, workload, financial compensation for program staff, reporting relationship, the #1 greatest challenge, and potential solutions.

### Limitations

We wish to note several limitations before putting the study in context and considering its implications. We should acknowledge that we may have overestimated the percentage of hospitals that have HCEPs since hospitals without such a program may have been less likely to have a “best informant” and therefore less likely to have participated in the study. However, there were no significant differences between participating hospitals and non-participating hospitals for any of the demographic variables examined in this study [23]. Another limitation is that among hospitals that said they had a HCEP, only one hospital without an ethics consultation service responded to the questions reported in this study. Thus while this study was designed to provide information on HCEPS in hospitals that provide ethics consultation as well as those that do not provide ethics consultation, the results provide almost no information about hospitals in the latter category. A further limitation relates to the completion of the survey by a single respondent or “best informant” at each hospital. Since in many hospitals, various ethics-related activities are carried out in silos in relative isolation from each other [5], the survey respondent may not have been aware of the full range of activities of their HCEP, especially if the program was not well integrated. An added challenge is that unlike EC, which has widely accepted definitions and standards, there are no widely accepted definition or standards for HCEPs. While we stipulated a definition for “health care ethics program” in the survey, the term has only recently been gaining in usage and acceptance with the trend towards adopting integrated ethics programs. In this survey, because we wanted to capture the full range of organized entities that support health care ethics, we deliberately defined HCEPs in a broad and general way. As such, the term was almost certainly interpreted differently by various respondents. Lastly, a limitation of the paper relates to the questions on salary support/compensation and our concerns about the validity of these results, because of both the number of non-respondents and the number of implausible responses observed for these questions.

### Prevalence, scope, and activities of HCEPs

Having acknowledged these limitations, we believe these data serve as the first systematically conducted survey of the state of HCEPs in US hospitals. It represents a starting point for further research and may serve to set a bar for future work. As such, we reflect on the study findings and their implications. The 97% prevalence of HCEPs in US hospitals is not surprising, considering that by 1999, 93% of hospitals had institutional ethics committees. We expected the prevalence of HCEPs to be quite high since HCEPs were defined to encompass all organized health care ethics activities including but not limited to ethics committees.

With regard to scope of HCEPs, our findings show that in almost all hospitals, HCEPs include the hospital’s clinical ethics functions, while other functions such as ethical leadership, research ethics, regulatory compliance, and business ethics were included in the scope of the HCEP in only a minority of hospitals. We should note that there are two possibilities for why hospitals do not report that the scope of their HCEP includes a particular function or content area: either that function is not carried out under the umbrella of the HCEP, or that function is not carried out at the hospital at all—at least not in an organized way. For example, a hospital might have an internal or external IRB that addresses aspects of research ethics but is separate from the hospital’s HCEP, or alternatively, the hospital might have no organized mechanism for addressing research ethics at all, which would likely be the case if they did not conduct research. While historically, most clinical research was conducted in academic medical centers, which comprise approximately 5% of hospitals in the U.S. [27], there has been a trend in favor of community hospitals conducting research [28], and now most health care facilities have at least one IRB [29]. In this study 12.6% of hospitals indicated that research ethics functions were within the scope of their HCEPs, but there may have been other hospitals that addressed research ethics separately from their HCEP.

With regard to the other content areas (ethical leadership, business ethics, regulatory compliance), only a minority of hospitals report that they are included in their HCEPs. While there is little doubt that all hospitals encounter ethical issues relating to all three of these content areas, it is unclear whether hospitals formally address ethics in these areas through programs or structures separate from their HCEPs, or not at all. As an example, all hospitals must meet regulatory compliance requirements [28]. Guidance from the U.S. Department of Health and Human Services Office of the Inspector General states that “it is imperative for hospitals to establish and maintain effective compliance programs” [30]. So, while only 29% of respondents reported that this activity resided with their HCEP, compliance activities are highly likely to be going on elsewhere in the hospital. Compliance programs are often called “Compliance and Ethics” programs, mirroring U.S. Sentencing Commission Guidelines for Organizations [31]. But it is unclear the extent to which compliance programs integrate both compliance-based and values- or integrity-based approaches to ethics [32].

HCEPs were much more likely to engage in certain activities compared to others. Most hospitals’ HCEPs were responsible for providing ongoing ethics education to all staff and to nurses, while a minority of hospitals were responsible for educating other target audiences. In terms of policy work, HCEPs most commonly reviewed existing policies and less often participated in developing new policies or assisting others in policy review. Most HCEPs had a representative in executive leadership, but less than half had representatives on other committees, led large-scale quality improvement initiatives relating to ethics, and engaged in community outreach.

### Extent of integration of HCEPs

Our findings pertaining to the scope and activities of HCEPs provide some preliminary insights into the degree to which HCEPs are integrated. Integrated ethics programs, by their nature, have a unified, coordinated structure; are comprehensive or at least very broad in scope, and utilize a variety of different strategies to integrate with other programs and individuals throughout the organization. In this survey we did not examine the extent to which HCEPs are unified or coordinated structurally, but we did ask questions about the breadth of HCEPs scope and activities. With respect to scope, we note that while in most hospitals the HCEP’s scope was rather narrow, in that it included only one or two of the listed content areas, in 11.8% of hospitals, the scope was broad, in that they included four or five of these areas. We also note that the mean number of content areas varied based on level of academic affiliation, suggesting that HCEPs in academic medical centers tend to be broader in scope.

We observed a similar pattern for HCEPs’ breadth of activities. In most hospitals the HCEP was responsible for educating only a few of the listed target audiences, but in 14.0% of hospitals the HCEP educated six or seven. In most hospitals the HCEP participated in only one or two of the listed types of policy work, but in 27.7% the HCEP participated in all four. And in most hospitals the HCEP performed only one of the other activities listed, but in 4.1% of hospitals, the HCEP participated in all four. For all of these variables—content areas, target audiences, types of policy work, and other activities, the mean number varied based on level of academic affiliation and location, suggesting that HCEPs in urban academic medical centers tend to be more integrated.

### Staffing, workload, and compensation for HCEP work

In reflecting on the study results regarding compensation for HCEP work, we must take into account that, with respect to both the number of individuals who received financial compensation and the estimated number of FTEs, there were a number of non-respondents to these questions and a number of participants gave responses that were considered implausible. This suggests that respondents may not have been sure about how to answer these questions and/or may have misinterpreted their intent. Hence any interpretation must be cautious. Nonetheless, a few observations deserve mention.

First, we observe that the estimated number of individuals who performed work for HCEPs in US hospitals in the year prior to the survey was 32,000, compared with 27,000 who performed ethics consultation [23]. This would suggest that 84% of individuals who work for HCEPs perform ethics consultation.

Second, it appears that large hospitals, major teaching hospitals, and urban hospitals devoted significantly more resources to HCEPs than other hospitals, in term of individuals working for the HCEP, the number of person hours spent on HCEP work, the number of individuals receiving financial compensation specifically for HCEP work, and the total number of FTEs. With respect to financial compensation, the differences were particularly striking: for example, the mean number of FTEs for hospitals with 500 or more beds and for major teaching hospitals was 1.0, compared with 0 for hospitals with fewer than 100 beds and for non-teaching hospitals.

In interpreting these results, it is important to note that the survey asked only about individuals who received financial compensation *specifically* for HCEP work in the form of, for example, a consulting fee or a dedicated percentage of their salary. Employees who performed work for the HCEP as a volunteer service activity, as a “collateral duty,” or as part of their administrative or protected time may have been 100% compensated for their HCEP work in the sense that they performed it while “on the clock,” even though they did not receive any compensation *specifically* for their HCEP work. In addition, while percent effort distribution (in which professionals have portions of their time allocated to specific activities) is a common practice in academic medical centers, this may be an unfamiliar concept in non-academic settings. As a result, this study may have underestimated financial compensation in these hospitals.

### Challenges and solutions

As we noted, while overall, a shortage of resources was the most commonly reported greatest challenge, in hospitals with fewer than 100 beds, the most commonly reported greatest challenge was underutilization. Responses categorized as underutilization included lack of staff awareness, understanding, or appreciation of the services offered by the HCEP, or lack of perceived need for the service. This finding of underutilization is consistent with the findings of a national survey of critical access hospitals conducted in 2007 [33]. In that survey, which included 381 hospital administrators, only 60% of these hospital administrators reported having an ethics committee or ethics consultation service and 28% did not see the need for such ethics support services despite their absence [33]. Is the challenge of underutilization at small hospitals concerning? It would seem to depend on whether health care professionals do in fact have the competence to address ethical concerns without collaborating with health care ethicists, and are doing so in a way that adequately and justly meets the needs of stakeholders at their hospitals. Our study cannot adequately determine whether that is the case, but it is an important issue to explore.

### Conclusion

Given the complicated mission and organizational structure of hospitals, and the many stakeholders involved, hospital leaders have a daunting task in meeting their mission in an ethically sound manner. Our study shows that nearly all US hospitals have some sort of HCEP, but these programs vary widely in terms of scope, activities, staffing, workload, and compensation of staff. Only a minority of HCEPs are integrated ethics programs in that they apply a unified, coordinated programmatic approach to managing ethics that address a broad range of ethics content areas and employ a wide range of strategies to integrate with other parts of the organization. The greatest challenge facing HCEPs is lack of resources, except in small hospitals where the greatest challenge is underutilization. We hope that our results will inform further discussion about the appropriate role of HCEPs and the resources they require in facilitating the delivery of ethically sound patient care in hospitals.

## Data Availability

The datasets generated and analyzed during this study may be requested from the authors. Because some survey data may reveal identifying information regarding participating hospitals, access to some data may be restricted.

## Abbreviations

ASBH: American Society for Bioethics and Humanities
CHA: Catholic Health Association
FTE: Full time equivalent
HCEP: Health care ethics program
IRB: Institutional Review Board

## Footnotes

1 Q1, Q41, Q47A-B, Q49-50, Q15, Q61-73, Q75-7.

2 Q1, Q47A, Q61, Q71, Q41, Q72.

## Declarations

### Disclaimer

The views expressed here are those of the authors and are not necessarily a reflection of the policies of their employers.
